# Measurement of change in health status with Rasch models

**DOI:** 10.1186/s12955-014-0197-x

**Published:** 2015-02-07

**Authors:** Pasquale Anselmi, Giulio Vidotto, Ornella Bettinardi, Giorgio Bertolotti

**Affiliations:** Department FISPPA, University of Padova, Via Venezia 8, 35131 Padova, Italy; Department of General Psychology, University of Padova, Via Venezia 8, 35131 Padova, Italy; Department of Mental Health and Pathological Addiction, Via delle Valli 5, 29121 Piacenza, Italy; Psychology Unit, Maugeri Foundation, Via Roncaccio 16, 21029 Tradate, VA Italy

**Keywords:** Measurement of change, Health status, Rehabilitation, Rasch, Item response theory

## Abstract

**Background:**

The traditional approach to the measurement of change presents important drawbacks (no information at individual level, ordinal scores, variance of the measurement instrument across time points), which Rasch models overcome. The article aims to illustrate the features of the measurement of change with Rasch models.

**Methods:**

To illustrate the measurement of change using Rasch models, the quantitative data of a longitudinal study of heart-surgery patients (*N* = 98) were used. The scale “Perception of Positive Change” was used as an example of measurement instrument. All patients underwent cardiac rehabilitation, individual psychological intervention, and educational intervention. Nineteen patients also attended progressive muscle relaxation group trainings. The scale was administered before and after the interventions. Three Rasch approaches were used. Two separate analyses were run on the data from the two time points to test the invariance of the instrument. An analysis was run on the stacked data from both time points to measure change in a common frame of reference. Results of the latter analysis were compared with those of an analysis that removed the influence of local dependency on patient measures. Statistics *t*, χ^2^ and *F* were used for comparing the patient and item measures estimated in the Rasch analyses (a-priori α = .05). Infit, Outfit, *R* and item Strata were used for investigating Rasch model fit, reliability, and validity of the instrument.

**Results:**

Data of all 98 patients were included in the analyses. The instrument was reliable, valid, and substantively unidimensional (Infit, Outfit < 2 for all items, *R* = .84, item Strata range = 3.93-6.07). Changes in the functioning of the instrument occurred across the two time, which prevented the use of the two separate analyses to unambiguously measure change. Local dependency had a negligible effect on patient measures (*p* ≥ .8674). Thirteen patients improved, whereas 3 worsened. The patients who attended the relaxation group trainings did not report greater improvement than those who did not (*p* = .1007).

**Conclusions:**

Rasch models represent a valid framework for the measurement of change and a useful complement to traditional approaches.

## Background

Accurate measurement of change in health status is an essential requirement for maintaining and improving the quality of health services. Such measurement is usually accomplished using a single group repeated measures design, where patients are assessed before and after an intervention. Change scores are computed for each patient by taking the difference between his/her scores in the two time points. The paired *t*-test statistic is often used to test the statistical significance of the change that has occurred over time, but it has the undesirable property of depending on the sample size. Effect-size statistics that remove such a dependence have therefore been developed. These statistics provide an estimate of the magnitude of change, standardized relative to the variability of change scores [1] or to the variability of baseline scores [2]. Values of .20, .50, and .80 or greater have been suggested to represent small, moderate, and large change, respectively [3].

The measurement of change based on the aforementioned approach presents important drawbacks. The *t*-test and the effect size statistics involve the mean change score and, therefore, they only measure the overall change of patients. No information is provided at the individual level. These statistics do not allow for a distinction between patients who responded to the intervention and those who did not, nor do they allow for a distinction between patients who responded with different degrees. At least two advantages derive from measuring the change at the individual level. First, the examination of patients who changed would allow for the identification of specific features of patients that are related to their likelihood of responding to the intervention. These features can then be used to identify *a priori* the patients who are good candidates for the intervention. Second, the intervention does not necessarily have to be the same for all patients, but it can differ. This is particularly useful in clinical research, where the number of patients who receive the same intervention is usually limited. A method for measuring the change at the individual level is desirable.

The scores that patients obtained on the measurement instrument are ordinal. Being ordinal, the unit difference between adjacent scores is not equal at different levels of the score domain. For example, a compression of the scale is bound to occur near the lower and upper boundaries of the domain (“floor” and “ceiling” effects, respectively) [4]. As a consequence, although it may be possible to determine whether change has occurred, it is hard to precisely quantify its extent. Interval measures are preferable to ordinal scores: They are characterized by measurement units that maintain the same size over the entire domain so that the measurement of change is more precise. Misusing ordinal scores as they were interval measures can lead to erroneous conclusions in clinical trials [5]. A method that produces interval measures from ordinal scores is desirable.

Patients are expected to change from Time 1 to Time 2 as a result of the intervention. However, the functioning of the measurement instrument might also change, even when identical collection protocols are used in the two time points. Some items are directly related to the intervention, whereas others are not. Thus, the intervention would affect most the responses to the former items. Moreover, patients might be quite impaired before the intervention, so the upper categories of the response scale (i.e., those indicating greater health) might be rarely used. After the intervention, patients might have made considerable improvement, so the lower categories (i.e., those indicating lower health) might be rarely used. Changes in the functioning of the instrument make the interpretation of change ambiguous [6]. A method is desirable that ensures the invariance of the instrument across time points.

One of the most promising approaches to the issue of the measurement of change is item response theory. Simple and convincing models within this framework are the Rasch models [7-9]. Rasch models characterize the responses of persons to items as a function of person and item measures. These measures pertain to the level of a quantitative latent trait possessed by a person or item, and their specific meaning relies on the subject of the assessment. In educational assessments, for instance, person measures indicate the ability of persons, and item measures indicate the difficulty of items. In health status assessments, person measures indicate the health of persons, and item measures indicate the severity of items. There is a long history of applications of Rasch models in medical field [10-16].

Rasch models overcome current drawbacks in the measurement of change. A measure is estimated for each patient so that the change can be measured at the individual level. The statistical significance of change is tested by means of the standard errors that characterize the measures. For Rasch analyses, if the data fit the model, interval measures are obtained from ordinal scores; this allows the measurement of change to be more accurate. Patients can be measured within a common frame of reference encompassing the different time points so that the measurement of change has an unambiguous numerical representation and a substantive meaning.

The article aims to illustrate the features of the measurement of change with Rasch models. Different Rasch-based approaches are described, and an illustrative application in the field of cardiac rehabilitation is presented.

## Methods

To illustrate the features of the measurement of change using Rasch models, the quantitative data of a longitudinal study of heart-surgery patients were used. The scale “Perception of Positive Change” of the Cognitive Behavioral Assessment - Outcome Evaluation (CBA-OE) [17,18] was used as an example of measurement instrument.

### Subjects

The sample consisted of 98 heart-surgery patients who were enrolled in a cardiac rehabilitation programme during hospitalization. Their mean age was 62.39 (*SD* = 10.03; range from 36 to 81), and 79 were male. Fifty-eight percent of patients completed up to 8 years of education, and 42% more than 8 years. Fifty-three percent are retired, 17% employed, 16% work on their own, 5% housewives, 2% unemployed, and 7% work occasionally. Eighty percent are married, 10% widowed, 4% separated or divorced, and 6% single. The study was approved by the local institutional review board (Salvatore Maugeri Foundation - IRCCS). All patients spontaneously gave their informed consent to participate in the study and to use the data. Patient records were anonymized and de-identified prior to analyses.

### Procedure

All patients underwent multidisciplinary cardiac rehabilitation, individual psychological intervention, and educational intervention, in accordance with guidelines [19,20]. Nineteen patients also attended progressive muscle relaxation group trainings, based on Jacobson’s method–reduced [21,22], as the psychologist requested.

The patients were assessed using the scale “Perception of Positive Change”. The scale consists of 11 items (see Table 1) evaluated on a 5-point scale (from “Not at all” - 0 to “Very much” – 4). Item 3 is a reverse item. The scale was administered shortly after hospitalization (Time 1), and shortly before discharge (Time 2). The time between the two assessments was about 3 weeks. Confusion might arise from the fact that the instrument used to measure change contains the word “Change” in the title. The scale measures the perception of being able to face difficulties, and of receiving support from others. It is measured the change of this perception from Time 1 to Time 2 as a result of the interventions.

**Table 1:** **The scale “Perception of Positive Change” of the Cognitive Behavioral Assessment - Outcome Evaluation (CBA-OE)** [17,18]

**Item no.**	**Item text**
1	I have felt supported by others
2	I have felt understood by others
3*	I have felt overcome by difficulties
4	I have felt able to react positively, even to difficulties and failures
5	I have felt the sensation that the worst was over
6	I have had the feeling of being sure of myself
7	I have seen possible solutions to my problems
8	I have managed to speak to others
9	I have tried to face difficulties rather than avoid them
10	Someone has helped me to solve my personal problems
11	I am satisfied with the goals I have achieved or I am about to achieve

*Reverse item.

### The measurement of change with Rasch models

A multitude of unidimensional clinical instruments are covered by three fundamental Rasch models. The simple logistic model (SLM) [7] is meant for dichotomous items (e.g., yes/no; present/absent), whereas the rating scale model (RSM) [23] and the partial credit model (PCM) [24] apply to polytomous items (e.g., never/sometimes/often/always; very difficult/difficult/easy/very easy). In the RSM, the response categories are defined identically for all items, whereas they are allowed to differ in the PCM (e.g., items with different number of response categories and/or different labels). The analysis results in a measure for each patient, indicating his/her health, and a measure for each item, indicating its severity. In the RSM and the PCM, measures are also estimated that describe the functioning of the response scale. These measures, called thresholds, represent the point on the latent variable where adjacent response categories are equally probable. The thresholds express the amount of the latent variable covered by each response category and, therefore, the probability of the response category itself.

The Rasch analysis starts from the *n* × *k* matrix *X* of the observed responses, where *n* is the number of patients and *k* is the number of items. Each cell of *X* contains the response *x*_*vi*_ of patient *v* to item *i*. In repeated measures designs, two matrices *X*_1_ and *X*_2_ contain the responses observed at Time 1 and Time 2, respectively.

A seemingly straightforward approach to the measurement of change would consist of running two separate Rasch analyses on *X*_1_ and *X*_2_. This approach (hereafter referred to as “separate analyses”) will provide two sets of patient, item, and threshold measures, one for each time point. The intra-patient differences between the patient measures could then be used as measures of individual change. Such an approach might not be feasible in practice. Between the two time points, not only the patients might have changed but also the functioning of the instrument. The intervention does not affect the responses to all items equally, but it more strongly influences the items it is directly related to. The use of the response categories might differ across the two time points as an effect of the different health statuses of the patients before and after the intervention. These changes would make the meaning of change uncertain.

For the measurement of change to have an unambiguous numerical representation and a substantive meaning, the patient measures should be estimated and compared within a common frame of reference encompassing both time points [6]. In such a frame of reference, instrument changes are controlled by fixing the item and threshold measures to be equal in the two time points. Two approaches are available [25,26]. In the first one, the data from a time point are analyzed to obtain the patient measures for that time point. Then, the data from the other time point are analyzed by anchoring the item and threshold measures to the values estimated in the previous analysis. This would provide a set of patient measures for the new time point, which are comparable with the previous ones. This approach requires the explicit identification of a time point as more decisive. If the emphasis is on making decisions about administering the intervention, Time 1 is more decisive, and then, it is measured at Time 1 and anchored at Time 2. If the emphasis is on making decisions about the outcome of the intervention (success, failure), Time 2 is more decisive, and then, it is measured at Time 2 and anchored at Time 1.

The second approach takes the more overall position that both time points are equally important. The data from the two time points are stacked on each other so that each item corresponds to one column and each time point for each patient is a row of the combined data set. The stacking of the two matrices *X*_1_ and *X*_2_ results in the 2*n* × *k* matrix *X*_1–2_. Estimating the Rasch model on the stacked data *X*_1–2_ (hereafter referred to as “stacked analysis”) provides a unique set of item and threshold measures that are consistent with both time points, and a patient measure for each patient in each time point.

In the stacked analysis, the patient measures at Time 1 and Time 2 might be influenced by local dependency across the two time points, if any exists. A simple approach for avoiding such an influence consists of the following steps [25-27]:

1. For each patient, the data for one of the two time points are selected at random so that each patient is in the selection only once but both time points are equally represented.
2. The Rasch analysis is run on the selected data. Given that, for each patient, only the data for one time point are considered, there will be no intra-patient dependencies across time points.
3. The Rasch analysis is run on the complete stacked data, with the item and threshold measures anchored at the values that were estimated on the selected data. The anchor values will prevent eventual dependency from distorting the patient measures at the two time points.

Hereafter, this approach will be referred to as “stacked analysis with anchors”. If the patient measures estimated in the stacked analysis with anchors do not differ from those estimated in the stacked analysis, then the effect of local dependency is negligible, and either one or the other measures can be used indifferently. Otherwise, the former measures should be used.

### Analysis procedure

The RSM was used because the response categories were the same for all items of the scale “Perception of Positive Change”. The analyses were run using the computer program Facets 3.66.0 [28]. Item 3 (the reverse item) was rescored prior to the analyses.

To investigate whether the functioning of the instrument differed across the two time points, two separate analyses were run on the data collected before and after the interventions. For each item *i*, the statistic ${t}_i=\left({\delta}_{i2}-{\delta}_{i1}\right)/\sqrt{S{E}_{i2}^2+S{E}_{i1}^2}$ was computed, where *δ*_*i*1_ and *δ*_*i*2_ are the measures of item *i* at Time 1 and Time 2, and *SE*_*i*1_ and *SE*_*i*2_ are the respective standard errors (*df* = 2*n* – 2). The thresholds (τ) and the probabilities of the response categories at the two time points were compared as well.

Then, a stacked analysis was run on the data from both time points. This approach was used because it provides a frame of reference for measuring change without having to consider one time point as more important than the other. The influence of local dependency was investigated by comparing the patient measures estimated in the stacked analysis with those estimated in a stacked analysis with anchors. For each patient *v* and each time point *t* ∈ {1, 2}, the statistic ${t}_{vt}=\left({\beta}_{vt}-{\beta}_{vt}^{*}\right)/\sqrt{S{E}_{vt}^2+S{E}_{vt}^{*2}}$ was computed, where *β*_*vt*_ and ${\beta}_{vt}^{*}$ are the measures of patient *v* at time *t* obtained on the stacked analysis and the stacked analysis with anchors, respectively (*df* = 2 *k* – 2).

To investigate whether the interventions have had the same effect on the patients, Pearson’s correlation between the patient measures at Time 1 and Time 2 was computed. The significance of change was tested at the individual level by computing, for each patient *v*, the statistic ${t}_v=\left({\beta}_{v2}-{\beta}_{v1}\right)/\sqrt{S{E}_{v2}^2+S{E}_{v1}^2}$, where *β*_*v*2_ and *β*_*v*1_ are the measures of patient *v* at Time 2 and Time 1, respectively (*df* = 2 *k* – 2). The significance of change was also tested at the group level by compounding the individual *p* values into the statistic χ^2^ = −2log(*p*_1_*p*_2_ … *p*_*n*_), with *df* = 2*n* [29]. Three statistics χ^2^ were computed, pertaining to 1) the entire group of patients; 2) the patients who only attended multidisciplinary cardiac rehabilitation, psychological, and educational interventions; and 3) the patients who also attended relaxation group trainings. To compare the magnitude of change in the last two groups, a statistic *F* was computed as the ratio between the statistics χ^2^ of the two groups divided by the respective *df*s (which are also the *df*s of *F*). For all significance tests, a-priori α was .05.

In all analyses, Rasch-based statistics were computed, that provide useful information about the fit of data to the Rasch model, the reliability, and validity of the instrument. Infit and Outfit mean-square statistics [30] are χ^2^ statistics divided by their degrees of freedom, with an expected value of 1. Outfit is more sensitive to unexpected responses on items which are far from person measure, whereas Infit is more sensitive to unexpected responses on items which are close to person measure. These statistics were computed for each patient and each item. Values larger than 2 for a particular patient suggest that he/she belong to a different population, or that he/she has filled out the scale inaccurately [28,31]. Infit and Outfit of the items provide evidence about the construct validity described by Messick [32]. Values greater than 2 for a particular item suggest that it is badly-formulated and confusing, or that it may measure a construct other than that measured by the other items (multidimensionality) [28,31]. The item Strata is also computed [33], which represents the number of statistically distinct groups of item measures that the patients have distinguished. If at least two groups are unable to be identified, then the variable defined by the items is hardly interpretable (low construct validity) [31]. Finally, the patient separation reliability *R* [33] was computed, which informs about reliability of the instrument. *R* is the Rasch equivalent of Cronbach alpha. It ranges from 0 to 1. The closer the value of *R* is to 1, the greater the probability that differences among the patient measures express actual differences among the patient health statuses.

## Results and discussion

The Rasch analyses were run on the data of all 98 patients. Infit and Outfit were smaller than 2 for all items. From 14 to 18 patients (out of 98) had Infit and/or Outfit greater than 2 at Time 1 and/or Time 2. Item Strata ranged from 3.93 to 6.07, and *R* was equal to .84. On the whole, these results suggest that the instrument was reliable, valid, and substantively unidimensional.

**Figure 1:**
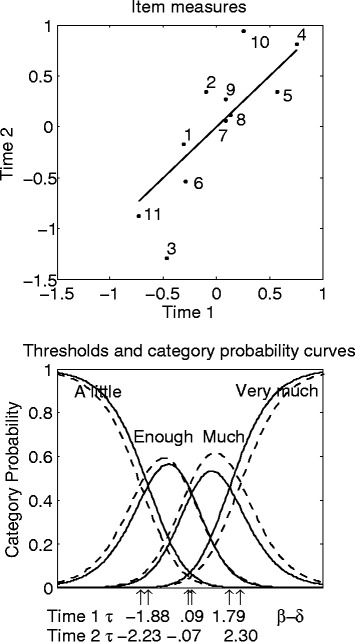
**Rasch analyses run separately on the data from the two time points.** The upper diagram depicts the item measures at Time 2 (*y* axis) plotted against those at Time 1 (*x* axis). Greater measures indicate more severe items. The identity line *x* = *y* is added for reference. The lower diagram depicts the thresholds and the probability curves of response categories at Time 1 (unbroken line) and Time 2 (broken line). “↑” points out the location of the thresholds on the latent variable.

This section presents the results of the two separate analyses that were run on the data from the two time points. The upper diagram of Figure 1 depicts the item measures at Time 2 (*y* axis) plotted against those at Time 1 (*x* axis). Greater measures indicate more severe items. Three out of 11 items are quite far from the identity line *x* = *y*. Items 2 and 10 were significantly more severe at Time 2 than at Time 1 (*t*_2_(194) = 2.01, *p* = .0458, Cohen’s *d* = .29; *t*_10_(194) = 3.10, *p* = .0022; Cohen’s *d* = .45), whereas Item 3 was significantly less severe (*t*_3_(194) = −3.50, *p* = .0006, Cohen’s *d* = .50). The lower diagram of Figure 1 shows the probability curves of response categories at Time 1 (unbroken line) and Time 2 (broken line). The patients have never used the response category “Not at all”, so in the present data the response scale goes from “A little” to “Very much”. In Rasch measurement, extreme response categories always approach a probability of 1 asymptotically because it is assumed that respondents with infinitely high (resp. low) measures must be observed in the highest (resp. lowest) categories regardless of the manner in which those categories are defined substantively or used by the sample [34]. At Time 1, the probability of responding “Enough” was slightly greater than that of responding “Much”, whereas at Time 2, the opposite occurred. The category “Enough” represented a greater amount of the latent variable than did the category “Much” at Time 1 (τ_A little-Enough_ − τ_Enough-Much_ = 1.97; τ_Enough-Much_ − τ_Much-Very much_ = 1.70), whereas it represented a lower amount at Time 2 (τ_A little-Enough_ − τ_Enough-Much_ = 2.16; τ_Enough-Much_ − τ_Much-Very much_ = 2.37). Moreover, the intermediate categories represented a wider range of the latent variable at Time 2 than at Time 1 (τ_A little-Enough_ − τ_Much-Very much_ = 4.53, 3.67 for Time 2 and Time 1, respectively). These changes in item severities and response category probabilities make the interpretation of change ambiguous.

This section presents the results of the stacked analysis and the stacked analysis with anchors. None of the patient measures that were estimated in the former analysis differed from those estimated in the latter (*p* ≥ .8674). Thus, in the present data, local dependency has had a negligible effect on patient measures. The patient measures obtained in the stacked analysis are considered in the following.

**Figure 2:**
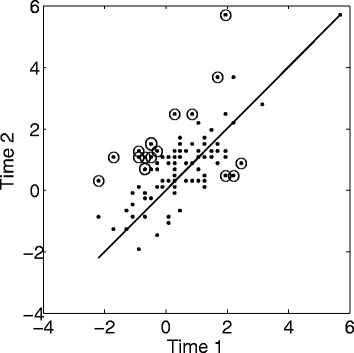
**Rasch analysis run on stacked data from the two time points.** The patient measures at Time 2 (*y* axis) are plotted against those at Time 1 (*x* axis). Greater measures indicate more healthy patients. Circled dots indicate statistically significant change. The identity line *x* = *y* is added for reference.

Figure 2 depicts the patient measures at Time 2 (*y* axis) plotted against those at Time 1 (*x* axis). Greater measures indicate more healthy patients. A moderate correlation is observed between the two measures (*r* = .67), meaning that the interventions did not affect the patients in a similar way. Thirteen patients reported a significant improvement from Time 1 to Time 2 (circled dots above the identity line), whereas 3 patients reported a significant worsening (circled dots below the identity line). Thus, the Rasch analysis provided information at the individual level, allowing the distinction between patients who have improved, worsened, or who have not changed. A significant improvement was observed in the entire group of patients (χ^2^(196) = 374.78, *p* < .0001); in the patients who only attended multidisciplinary cardiac rehabilitation, psychological, and educational interventions (χ^2^(158) = 283.48, *p* < .0001); and in those who also attended relaxation group trainings (χ^2^(38) = 91.30, *p* < .0001). The patients who attended the relaxation group trainings did not report greater improvement than the patients who did not (*F*(38, 158) = 1.34, *p* = .1007).

## Conclusions

Rasch models represent a valid framework for the measurement of change and a useful complement to traditional approaches. In the present study, the change has been measured at the individual level as well as in groups of patients who received different interventions. Patients have not been investigated with the aim of identifying the specific features of those who improved, worsened, or did not change. Future investigation will be devoted to this purpose. In the present study, precision and meaning of the measurement were derived from the interval level of the measures and the invariance of the instrument across time points. However, some patients did not fit the Rasch model, so that the validity of their measure is questionable. Further investigation is needed to understand the causes of misfit (Do these patients belong to a different population? Do they have filled out the scale inaccurately). Rasch models are especially demanding of data that satisfy the requirements for constructing measures. Two alternative pathways can be pursued when the data do not fit a Rasch model [35]. The first one consists of modifying the instrument, the definition of the construct under investigation, or both, in order to generate new data that better conform to the model. The second one consists of identifying an alternative model, usually within the framework of item response theory, that accounts better for the given data.

Responsiveness is an instrument’s ability to detect change [36]. Research on responsiveness generally presents the patients with a battery of instruments before and after a well-known efficacious intervention and then compares their responsiveness through some indexes which are based on the measurement of patient change. Highly responsive instruments are chosen for applications in clinical trials. Different indexes may provide different rank orderings of instrument responsiveness [37]. By taking into account aspects concerning the patients, the items, and the response scale, the Rasch models might provide a relevant contribution to the investigation of responsiveness.

There are other Rasch methods to the measurement of change [38-42], that have not been taken into account in the present study. Future studies should compare them in health fields experiencing different degrees and direction of change.

## Abbreviations

CBA-OE: Cognitive Behavioral Assessment - Outcome Evaluation
SLM: Simple Logistic Model
RSM: Rating Scale Model
PCM: Partial Credit Model
